# Generation of Recombinant Schmallenberg Virus Nucleocapsid Protein in Yeast and Development of Virus-Specific Monoclonal Antibodies

**DOI:** 10.1155/2014/160316

**Published:** 2014-05-29

**Authors:** Justas Lazutka, Aurelija Zvirbliene, Indre Dalgediene, Rasa Petraityte-Burneikiene, Aliona Spakova, Vilimas Sereika, Raimundas Lelesius, Kerstin Wernike, Martin Beer, Kestutis Sasnauskas

**Affiliations:** ^1^Institute of Biotechnology, Vilnius University, V.A. Graiciuno 8, 02241 Vilnius, Lithuania; ^2^Institute of Microbiology and Virology, Veterinary Academy, Lithuanian University of Health Sciences, Tilzes 18, 47181 Kaunas, Lithuania; ^3^Institut für Virusdiagnostik, Friedrich-Loeffler-Institut, Suedufer 10, 17493 Greifswald-Insel Riems, Germany

## Abstract

Schmallenberg virus (SBV), discovered in continental Europe in late 2011, causes mild clinical signs in adult ruminants, including diarrhoea and reduced milk yield. However, fetal infection can lead to severe malformation in newborn offspring. To develop improved reagents for SBV serology, a high-level yeast expression system was employed to produce recombinant SBV nucleocapsid (N) protein. Recombinant SBV N protein was investigated as an antigen in SBV-specific IgG enzyme immunoassay and used for generation of monoclonal antibodies (MAbs). Yeast-expressed SBV N protein was reactive with anti-SBV IgG-positive cow serum specimens collected from different farms of Lithuania. After immunization of mice with recombinant SBV N protein, four MAbs were generated. The MAbs raised against recombinant SBV N protein reacted with native viral nucleocapsids in SBV-infected BHK cells by immunofluorescence assay. The reactivity of recombinant N protein with SBV-positive cow serum specimens and the ability of the MAbs to recognize virus-infected cells confirm the antigenic similarity between yeast-expressed SBV N protein and native viral nucleocapsids. Our study demonstrates that yeast expression system is suitable for high-level production of recombinant SBV N protein and provides the first evidence on the presence of SBV-specific antibodies in cow serum specimens collected in Lithuania.

## 1. Introduction

In 2011, an unidentified disease in cattle was first reported in Germany in a farm near the town of Schmallenberg [1]. Metagenomic analysis identified a novel* Orthobunyavirus*, which subsequently was isolated from blood specimens of infected animals. This new virus was called Schmallenberg virus (SBV) after the place of origin of the collected samples. Clinical symptoms of diseased cows include fever, reduced milk yield, and diarrhoea. Also, SBV infection has been implicated in many cases of severely malformed bovine and ovine offspring [2–7]. The inoculation of 9-month-old calves with blood of cattle that were RT-qPCR positive for SBV caused fever and mucous diarrhoea, providing experimental evidence that SBV might be responsible for the clinical signs observed [1]. Analysis of viral genomic sequences has led to the classification of SBV in the Bunyaviridae family and the* Orthobunyavirus* genus. Recent analysis revealed that SBV is most related to Douglas and Sathuperi virus belonging to the Simbu serogroup of* Orthobunyavirus* genus [8]. The majority of bunyaviruses are transmitted by arthropod vectors. Epidemiological data existing so far are in accordance with the hypothesis that SBV is transmitted by biting midges (*Culicoides* spp.). Recently, some studies have reported the presence of the SBV genome in different species of* Culicoides* collected in different countries of Europe. It has been reported that some* Culicoides* species are present inside farm buildings during the winter and are able to complete their life cycle in animal enclosures. It is possible that SBV is able to persist from year to year in the vector population despite winter temperatures as described in reviews [6, 7]. The qRT-PCR is the primary diagnostic assay used by laboratories in affected countries [1]. This assay has limitations in detecting infected individuals based on blood samples, as it only detects viral RNA when the animal is viraemic [9]. Furthermore, the virus can be isolated on insect and hamster cell lines. For the detection of SBV-specific antibodies, indirect immunofluorescence tests, microneutralization tests, and commercial SBV-based indirect ELISA have been used [9–12].

The genetic structure of SBV is typical for Bunyaviridae, containing a tripartite RNA genome of negative polarity. The genome of SBV contains three segments of single-stranded negative-sense RNA called the large (L), medium (M), and small (S) segments. The L segment encodes the RNA-dependent RNA polymerase; M segment encodes surface glycoproteins Gn and Gc and nonstructural protein NSm. The S segment encodes nucleocapsid protein N and nonstructural protein NSs [13]. The S segment of SBV was shown to share 96.7% nucleotide sequence similarity with S segment of Shamonda virus. Comparably, the similarity between SBV and Sathuperi virus S segment nucleotide sequence is 94% [14].

The N protein of bunyaviruses is the most abundant viral antigen present in the virion and in the infected cells, thus making it an excellent target for serology [15–17]. Recombinant N proteins of different hantaviruses, generated in* Escherichia coli, *insect cells, or yeast have been widely used for serological diagnosis of hantavirus-specific antibodies in human sera and oral fluid [18–22].

The synthesis of N proteins of different European, Asian, and American hantaviruses in yeast expression system has been shown to result in large yields. The proteins were highly pure after nickel chelation purification and during stable long-term storage. Moreover, the recombinant hantavirus N proteins were strongly immunogenic in rabbits and mice. The yeast-expressed N proteins of different hantaviruses have been employed to develop highly sensitive and specific ELISAs and immunoblot tests [17–22]. Initial studies based on* E. coli* expression systems for hantavirus diagnostics have demonstrated lower specificities of these tests due to* E. coli* contaminants remaining in recombinant protein preparation [23, 24]. These problems were eliminated using yeast expression system [17–22].

Epidemiologic situation in regard to SBV infection may differ greatly from country to country and warrants further study. Indeed, to determine the true occurrence and prevalence of the SBV infection, fast, convenient, and cheap diagnostic tests are needed. In the current study, we have generated the N protein of SBV in yeast expression system, demonstrated its antigenic similarity with viral N protein, and developed N protein-specific MAbs reactive with SBV in infected cells.

## 2. Materials and Methods

### 2.1. Strains, Media, Yeast Transformation, and Cultivation

Recombinant construct containing SBV N gene sequence was amplified in* E. coli* DH5*α*F′ cells.* Saccharomyces cerevisiae* AH22-214* MATa* (*leu2 his4 pep4*) was used for expression of SBV N protein. Selection of yeast transformants resistant to formaldehyde was carried out on the YEPD (1% yeast extract, 2% peptone, and 2% dextrose) agar supplemented with 5 mM formaldehyde.* S. cerevisiae* transformants were grown in YEPD medium supplemented with 5 mM formaldehyde or in YEPG induction medium (1% yeast extract, 2% peptone, and 2,5% galactose) as described previously [25].

### 2.2. Cloning of SBV N Protein-Encoding Sequences into Yeast Vectors and Purification of Recombinant N Protein from Transformed Yeast

All DNA manipulations were performed according to standard procedures [26]. Enzymes, molecular mass standards, and kits for DNA manipulations were purchased from Thermo Fisher Scientific Baltics (Vilnius, Lithuania). SBV N gene was chemically synthesized by GenScript USA Inc. (Piscataway, NJ, USA) according to the published sequence GenBank accession number HE649914.1 [1]. The XmaJI sites compatible with XbaI site for cloning into yeast expression vectors were inserted into the ends of SBV N gene during gene synthesis. For the generation of N-terminally hexahistidine-tagged SBV N protein, the gene was cloned into XbaI site of the* S. cerevisiae* expression vector FX7-6-His under control of galactose inducible* S. cerevisiae GAL10* promoter described previously [17]. The resulting plasmid pFX7-SBV-6-HisN was used for transformation of yeast* S. cerevisiae* AH22-214, as described previously [25]. The primary structure of the cloned gene was confirmed by sequencing.

Cultivation of transformed yeast cells as well as the expression and purification of recombinant proteins was performed as previously described [17, 25]. Briefly, 100 mL of YEPD growth medium (yeast extract 1%, peptone 2%, and glucose 2%) supplemented with 5 mM formaldehyde was inoculated with the transformed yeast cells and grown with shaking at 30°C for 24 h. After addition of 100 mL of YEPG induction medium (yeast extract 1%, peptone 2%, and galactose 5% supplemented with 5 mM formaldehyde), the yeast cells were grown for additional 17 h. The cells were harvested, washed with distilled water, and frozen at −20°C until further use. Thawed cells were resuspended in 8 mL of disruption buffer (6 M guanidine hydrochloride, 0.1 M NaH_2_PO_4_, 0.5% glycerol, 1% Tween-20, 10 mM imidazole, 2 mM PMSF, and pH 8.0) and 8 g of glass beads (0.5 mm diameter, Sigma-Aldrich Co., St. Louis, MO, USA) was added. Cells were disrupted by vortexing at 4°C for 5 min. The cell debris was sedimented by centrifuging the obtained yeast lysates at 3000 ×g for 5 min. Insoluble proteins were spun down by centrifugation at 10.000 ×g for 10 min at 4°C. The supernatant was mixed with 2 mL of Ni-NTA resin equilibrated in disruption buffer and binding was performed by shaking for 1 h at room temperature (RT). N protein purification was performed on a polypropylene column, according to the manufacturer's recommendations for denaturing purification of insoluble proteins (Qiagen, Hilden, Germany). The main portion of the protein was eluted in buffer E (8 M urea, 0.1 M NaH_2_PO_4_, 0.01 M Tris, and pH 4.5). To ensure the purity of the eluted recombinant N protein SDS-PAGE, Coomassie Brilliant Blue staining and western blot were performed. Eluted protein was dialysed against sodium acetate buffer (50 mM sodium acetate, 100 mM sodium chloride, and pH 5.0) and stored at −20°C with 40% of glycerol. Yeast transformant containing the plasmid pFX7-6-His without any insert was used as a negative control.

### 2.3. SDS-PAGE and Western Blot Analysis

Protein samples were boiled in a reducing sample buffer and separated in a SDS-Tris-glycine buffer through polyacrylamide gel electrophoresis (PAGE). Proteins were visualized by staining with Coomassie Brilliant Blue (Sigma-Aldrich Co.). For western blot, purified proteins were electrotransferred to Roti-PVDF membrane (Carl Roth GmbH & Co., Karlsruhe, Germany). The membrane was blocked with RotiBlock (Carl-Roth GmbH & Co.) blocking solution for 2 h at RT and rinsed in PBS with 0.1% Tween-20 (PBST). The membrane was then incubated for 1 h at RT with primary antibodies at working dilution in PBST with 10% RotiBlock and subsequently incubated with goat anti-mouse IgG (H+L)-HRP conjugate (Bio-Rad, Hercules, CA, USA) 1 : 4000 diluted in PBST with 10% RotiBlock. The enzymatic reaction was developed using 4-chloro-1-naphthol and H_2_O_2_ (Fluka, Milwaukee, WI, USA). For the analysis of MAb specificity, undiluted hybridoma supernatants were used. To check the purity of recombinant N protein, MAb against 6-His-tag epitope (Thermo Scientific, Rockford, IL, USA) was used as a primary antibody.

### 2.4. Indirect Enzyme-Linked Immunosorbent Assay (ELISA) for Investigation of SBV N Protein-Specific Mouse Antibodies

Micro test plates (Nerbe Plus GmbH, Winsen/Luhe, Germany) were coated with 100 *μ*L/well of SBV N protein dissolved in the coating buffer (0.05 M sodium carbonate, pH 9.5) to a concentration of 5 *μ*g/mL. The plates were incubated overnight at 4°C. The coated plates were blocked with 250 *μ*L/well of PBS with 2% BSA for 1 h at RT. Then plates were rinsed twice with PBST. Mouse antiserum samples, hybridoma growth medium, or polyclonal antibodies were diluted in PBST, added to the wells (100 *μ*L/well), and incubated for 1 h at RT. The plates were then incubated for 1 h with goat anti-mouse IgG (H+L)-HRP conjugate (Bio-Rad) 1 : 5000 diluted in PBST. The enzymatic reaction was visualized by the addition of 100 *μ*L of “NeA-Blue” TMB solution (Clinical Science Products Inc., Mansfield, MA, USA) to each well. The reaction was stopped by adding 50 *μ*L/well of 1 M H_2_SO_4_ solution. The optical density (OD) was measured at 450 nm (reference filter 620 nm) in a microplate reader (Sunrise Tecan, Männedorf, Switzerland). The apparent dissociation constants (*K*_*d*_) of MAbs were determined by an indirect ELISA. The* K*_*d*_ values were calculated from 4 parallel ELISA titration curves fitting logistic model obtained by incubating plate-coated SBV N protein with increasing amounts of MAbs ranging from 1.9 × 10^−13^ M to 3.3 × 10^−8^  M. The SD was determined from these 4 calculated* K*_*d*_ values. The* K*_*d*_ for each MAb was defined as the concentration (M) of the MAb that gives one-half of the maximum OD_450_ value.

### 2.5. Indirect IgG ELISA for Detection of SBV N Protein-Specific Antibodies in Cow Serum

Cow blood samples were collected in May-June 2013 from cow farms located in different places of Lithuania. The sera were tested for antibodies against SBV using a commercially available ELISA kit (ID Screen Schmallenberg virus Indirect, IDvet, Grabels, France) [11] before testing them with recombinant SBV N protein. In the current study, 102 serum samples were used from this collection for the evaluation of yeast-derived recombinant SBV N protein as an antigen for ELISA. Micro test plates (Nerbe Plus GmbH) were coated with 400 ng per well of recombinant SBV N protein in 100 *μ*L of 0.05 M carbonate-bicarbonate coating buffer (pH 9.6) and incubated overnight at 4°C. Plates were washed three times with PBST and then blocked by the addition of 150 *μ*L of blocking reagent per well (1x Roti-Block, Carl Roth GmbH & Co.). The plates were incubated at RT for 1 hour. After blocking, the plates were washed three times with PBST and 100 *μ*L aliquots of serum specimens, 1 : 200 diluted in PBST buffer with 10% RotiBlock (Carl Roth GmbH & Co.), was added to the wells. Plates were incubated for 1 h at 37°C and washed five times with PBST. 100 *μ*L aliquots of rabbit antibovine IgG (Sigma-Aldrich Co.) conjugated to HRP, 1 : 10.000 (v/v) diluted in PBS with 5% RotiBlock, was added to each well and the plates were incubated for 1 h at 37°C. After washing five times with PBST, 100 *μ*L of TMB substrate (Clinical Science Products) was added to each well and the enzyme reaction was stopped with an equal volume of 1 M H_2_SO_4_ solution. The optical density at 450 nm was determined for each sample using an ELISA plate reader (Sunrise Tecan).

### 2.6. Mass Spectrometric Analysis of Recombinant Proteins

Mass spectrometric (MS) analysis of recombinant SBV N protein was carried out according to Hellman et al. [27]. Proteins were identified by matrix-assisted laser desorption/ionization (MALDI) mass spectrometry using a 4800 MALDI TOF/TOF mass spectrometer (Applied Biosystems/MDS SCIEX 4800 MALDI TOF/TOF, Framingham, MA, USA). Peptide mass spectra were acquired in reflector positive ion mode with a* m*/*z* range of 800–4000 Da. Four hundred laser shots were summarized for each sample with a mass accuracy of ±50 ppm. MS/MS spectra for dominating peptides were acquired in positive mode with the ion collision energy set to 1 keV. Five hundred laser shots were accumulated for each spectrum with a mass accuracy of ±0.1 Da. The proteins were identified in the TrEMBL database (3-23-10 release) using the Mascot algorithm.

### 2.7. Production of Monoclonal Antibodies

MAbs to recombinant SBV N protein were produced essentially as described by Kohler and Milstein [28]. Eight-week-old female BALB/c mice (obtained from a breeding colony at the Center for Innovative Medicine, Vilnius, Lithuania) were immunized at days 0, 28, and 56 by a subcutaneous injection of 50 *μ*g of recombinant SBV N protein. For a primary immunization, the antigen was emulsified in complete Freund's adjuvant (Sigma-Aldrich Co.). The second and the third immunisations were performed with the antigen dissolved in PBS. Antiserum samples were collected on the 14th day after the first, second, and third immunizations and tested by an indirect ELISA for the presence of IgG antibodies specific to SBV N protein. Three days after the final injection, mouse spleen cells were fused with Sp2/0-Ag 14 mouse myeloma cells using polyethylene glycol 1500 (PEG/DMSO solution, HybriMax, Sigma-Aldrich Co.). Hybrid cells were selected in growth medium supplemented with hypoxanthine, aminopterin, and thymidine (50x HAT media supplement, Sigma-Aldrich Co.). Samples of supernatant from wells with viable clones were screened by an indirect ELISA as described above. Hybridomas secreting specific antibodies to SBV N protein were subcloned twice by a limiting dilution assay. Hybridoma cells were maintained in complete Dulbecco's modified Eagle's medium (DMEM, Biochrom, Berlin, Germany) containing 15% fetal calf serum (Biochrom) and antibiotics. Antibodies in hybridoma culture supernatants were isotyped using the BD Pharmingen Mouse Immunoglobulin Isotyping ELISA Kit (BD Bioscience, San Diego, CA, USA) in accordance with the manufacturer's protocol. All procedures involving experimental mice were performed under controlled laboratory conditions in strict accordance with the Lithuanian and European legislation.

### 2.8. Immunofluorescence Assay

The immunofluorescence test was performed using SBV-infected BHK cells, clone BRS5 (L194, Collection of Cell Lines in Veterinary Medicine, Greifswald-Insel Riems) as antigen matrix in accordance with the procedure described previously [11]. Briefly, a cell suspension was seeded, incubated for 24 h at 37°C, and subsequently infected with SBV strain BH80/11. Forty-eight hours after infection the medium was removed and the cells were fixed using heat treatment (2 h at 80°C). Both, infected and uninfected cells were incubated with each MAb (1 : 10 diluted hybridoma supernatants) for 1 h at RT. After washing with Tris-buffered saline with 0.1% Tween-20 (TBST), a fluorescein isothiocyanate (FITC-) conjugated goat anti-mouse IgG (Sigma-Aldrich Co.) was added and incubated for 1 h at RT. Thereafter, the cells were washed, embedded with Dabco fluorescence conservation buffer (Sigma-Aldrich Co.), and analyzed using an inverted fluorescence microscope (Nikon Eclipse Ti-U, Nikon Instruments Inc., Melville, NY, USA).

## 3. Results and Discussion

### 3.1. Synthesis of SBV N Protein in Yeast* Saccharomyces cerevisiae*

**Figure 1:**
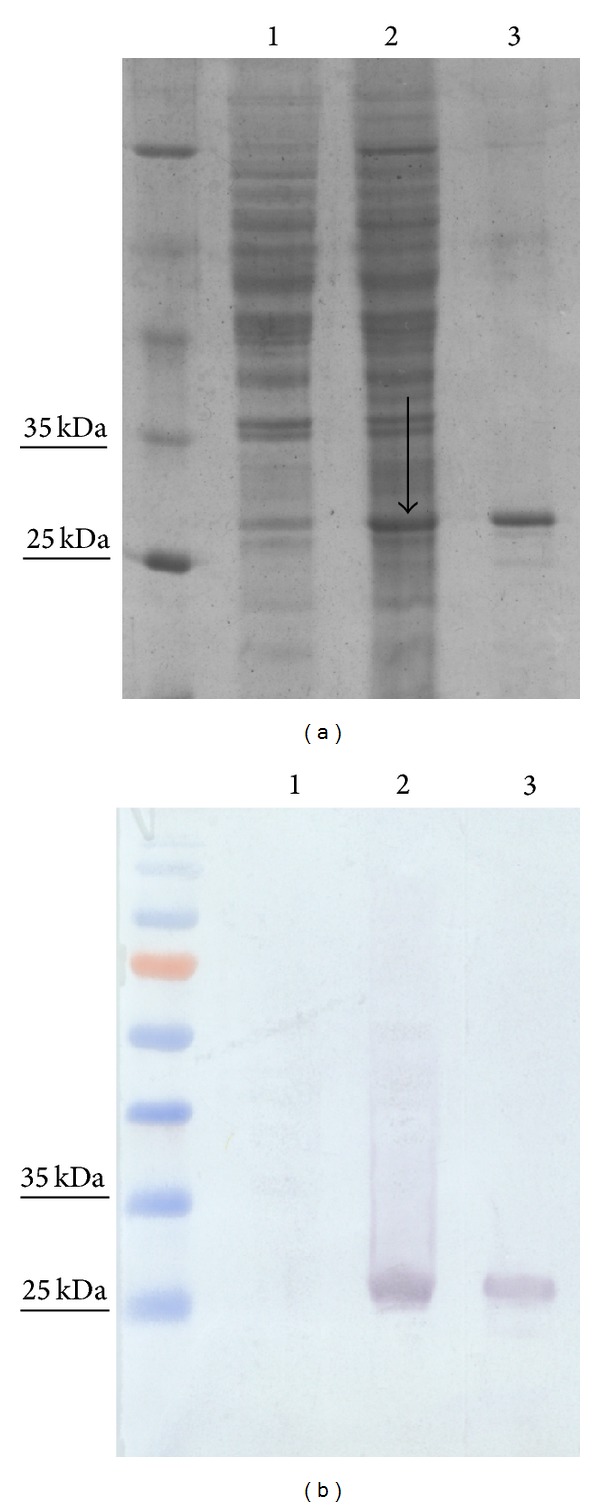
Analysis of yeast cell lysates and purified SBV N protein by SDS-PAGE (a) and western blot (b). Purified SBV N protein (lane 3) or 20 *μ*g of yeast lysates (lanes 1 and 2) was separated in a 12% SDS-PAGE gel and stained with Coomassie Brilliant Blue. PageRuler Unstained Protein Ladder (Thermo Fisher Scientific Baltics) was used. Lane 1, lysate of mock-transformed* S. cerevisiae* [pFX7-6-His]; lane 2, lysate of* S. cerevisiae *transformed with a plasmid [pFX7-6-His-N] encoding SBV N protein (the arrow indicates SBV N protein band); lane 3, Ni-chelate resin-purified SBV N protein. Western blotting was performed using the MAb against 6-His-tag epitope (b) (Thermo Scientific). PageRuler Prestained Protein Ladder (Thermo Fisher Scientific Baltics) was used.

To express the N protein of SBV virus, we exploited yeast vector system previously used for the high-level expression of hantaviruses N proteins [17]. Expression efficiency of recombinant SBV N protein in yeast was proven both by electrophoresis and immunoblotting. SDS-PAGE analysis of crude lysates of* S. cerevisiae* harbouring pFX7-SBV– expression vector revealed the presence of an additional protein band after induction with galactose. This band of approximately 26 kDa was present in the lysates of yeast transformed with pFX7-SBV-6-His-N (Figure 1(a), lane 3). Meanwhile, no additional band of the corresponding molecular size was observed in crude lysates of* S. cerevisiae* harbouring pFX7-6-His vector used as a negative control (Figure 1(a), lane 1). As evaluated by SDS-PAGE, the expression level of SBV N protein was approximately 2% of the total cellular protein. After cell lysis, the main quantity of SBV N protein was found in the insoluble fraction (data not shown). The yield of the His-tagged N protein after nickel-chelate chromatography was about 3.0–3.5 mg/g wet weight of yeast. Recombinant SBV N protein was soluble in 50 mM sodium acetate buffer (pH 5.0) containing 100 mM sodium chloride. The eluted N protein was visible as a single protein band in Coomassie Brilliant Blue-Stained SDS-PAGE gels (Figure 1(a)). In order to confirm the sequence identity of the full-length recombinant SBV N protein and determine its molecular weight, enzymatic digests were performed using trypsin, chymotrypsin, and endopeptidase-AspN to generate internal peptides for detailed mass spectrometry (MS) analysis. Peptide analysis confirmed the primary structure of recombinant SBV N protein predicted from DNA sequence (data not shown). Also, MS analysis revealed that the molecular weight of SBV N protein is 26 kDa, which is in line with theoretical calculated molecular weight of the protein. Western blot analysis of purified protein with the MAb against 6-His-tag epitope confirmed its identity (Figure 1(b), lane 3).

These data confirmed that we have constructed an efficient recombinant yeast expression system and obtained high-level expression of SBV N protein in yeast* S. cerevisiae*.

### 3.2. The Reactivity of Yeast-Derived SBV N Protein with SBV IgG-Positive Cow Sera

The reactivity of yeast-expressed SBV N protein with cow serum IgG induced by a natural SBV infection was analysed by an indirect IgG SBV ELISA using a panel of 102 cow serum specimens found to be either positive or negative for SBV-specific IgG antibodies using a commercial diagnostic kit (ID Screen Schmallenberg virus Indirect, IDvet). In the commercial kit, SBV N protein expressed in* E. coli* is used [11]. To define the positive/negative threshold of the newly developed indirect IgG SBV ELISA, 11 serum samples previously determined as SBV IgG-negative by commercial ID Screen test were used. The OD values of ELISA were corrected for nonspecific reactivity and reported as sample-to-positive (S/P) values (S/P = (OD sample/OD positive control (from the commercial test kit))∗100) according to Breard et al. [11]. To calculate the cut-off value, the reactivities of positive and negative reference serum samples from the ID screen test kit with the recombinant SBV N protein were analysed. The cut-off value was calculated as the mean of S/P values of negative samples plus 2 SD (22 + 10) with 95% confidence. Serum sample was considered positive when its S/P value was greater than 32. The tests were run in duplicate. The correlation coefficient and the standard error were 0.92 and 0.1, respectively, between separate runs. To prove the specificity of the newly developed indirect IgG SBV ELISA, yeast-expressed hantavirus Andes N protein as a negative control antigen was used [29]. No reactivity of cow serum specimens (OD at 450 nm was 0.15 or S/P = 11) with the recombinant hantavirus N protein was detected (data not shown).

**Figure 2:**
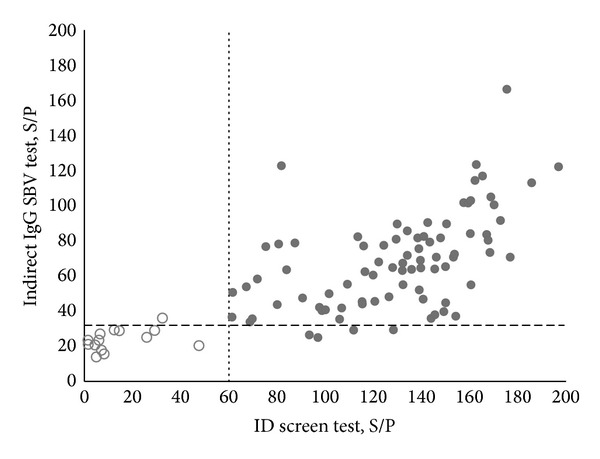
Antibody responses of individual cow serum specimens defined by the newly developed indirect IgG SBV ELISA based on yeast-expressed SBV N protein in comparison to the commercial ID screen test. The S/P ratios of reactivity were plotted. Grey markers represent positive serum samples and white represent negative serum samples obtained by commercial ID screen test. The dashed line represents the cut-off value of the newly developed indirect IgG SBV ELISA. The dotted line represents the cut-off value of the commercial ID screen test.

Seventy-eight serum specimens out of 82 samples positive by the commercial test were positive and 4 were negative by the newly developed indirect IgG SBV ELISA (Figure 2). Therefore, the sensitivity of the indirect IgG SBV ELISA was calculated to be 95% (78/82 × 100). Fourteen serum samples out of 15 negative specimens by the commercial test were negative and one was positive by the indirect IgG SBV ELISA (Figure 2). Thus, the specificity of the newly developed indirect IgG SBV ELISA was 93% (14/15 × 100). Five serum specimens gave doubtful results by the commercial test. The doubtful serum specimens obtained by the commercial test were excluded from the calculation, as some authors exclude undefined or grey zone sera from the analysis [30, 31].

The high number of sera from SBV-infected cows that were found to be positive in the newly developed indirect IgG SBV ELISA indicated that yeast-expressed SBV N protein may be a suitable antigen for serological diagnosis of SBV infection in cows. The observed discrepancies with the commercial test suggest that IgG SBV ELISA should be further evaluated and optimized using more cow serum specimens collected at variable intervals of the course of the disease. Mansfield and colleagues [12] have shown that the commercial ELISA test could not recognize all SBV-positive serum samples. They conducted focus reduction neutralization assay (PRNT) that appeared to be more sensitive than the commercial ELISA. The essential difference between the PRNT assay and SBV N protein-based ELISA is that the PRNT assay allows detection of antibodies against all structural viral proteins. These results suggest that serologic ELISA tests might be improved by incorporating other viral structural proteins in the test.

This is the first report on SBV antigen expression in yeast* S. cerevisiae* and on the development of an indirect IgG ELISA test based on yeast-expressed SBV N protein. The results showed comparable agreement with commercially available test based on* E. coli-*expressed SBV N protein [11] indicating that yeast-expressed SBV N protein could provide an alternative for analyzing SBV-specific antibodies in blood sera of SBV-infected cows.

The reactivity of yeast-expressed SBV N protein with SBV IgG-positive cow sera is in line with previous studies that demonstrated the usefulness of recombinant viral N proteins expressed in different heterologous systems for serological diagnosis of bunyaviruses infection both by western blot and ELISA [17–21]. The N protein of bunyaviruses is the most abundant viral antigen present in the virion and in the infected cells, thus making it an excellent target for serology as well as epidemiological studies of viral infections [16–18]. Recombinant N protein of hantaviruses, generated in* E. coli*, insect cells, or yeast, has been widely used for detection of hantavirus-specific antibodies in human sera and oral fluid [17–21]. SBV N protein was expressed recently in* E. coli* [11, 32] and used for the development of a commercial diagnostic kit for serologic diagnosis of SBV infection [11].

Taken together, the reactivity of SBV N protein with cow sera suggests that yeast-derived SBV N protein represents a suitable antigen for serologic detection of SBV infection and generation of virus-specific MAbs.

### 3.3. Generation of Monoclonal Antibodies against Recombinant SBV N Protein

**Figure 3:**
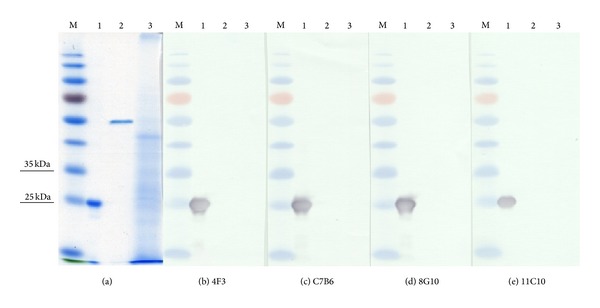
SDS-PAGE (a) and western blot analysis of recombinant SBV N protein with SBV N-specific MAbs ((b)–(e)). Lane M, PageRuler Prestained Protein Ladder (Thermo Fisher Scientific Baltics); lane 1, SBV N protein; lane 2, Puumala N 6 His-tagged protein; lane 3, yeast cell lysate. Undiluted hybridoma culture supernatants were used. MAbs codes are indicated at the bottom of each picture.

Purified recombinant SBV N protein was used to immunize mice and generate SBV N protein-specific MAbs. After screening and cloning of positive hybridoma clones, four stable hybridoma cell lines producing IgG antibodies were derived: 4F3, 7B6, 8G10, and 11C10. Hybridoma clones 7B6, 8G10, and 11C10 produced MAbs of IgG1 subtype, whereas the MAb produced by clone 4F3 was of IgG2b subtype (Table 1). The apparent* K*_*d*_ of the MAbs ranged between 2,  47 × 10^−9^ and 1,  39 × 10^−10^  M, which indicates high-affinity binding (Table 1). All MAbs reacted specifically with recombinant SBV N protein and did not react with yeast-expressed N proteins of Puumala, Hantaan, or Dobrava-Belgrade viruses [17] used to investigate their cross-reactivity (data not shown). To characterize the nature of the epitopes recognized by the MAbs, their reactivity in western blot was analyzed. All four MAbs recognized SDS-denatured SBV N protein in western blot assay (Figures 3(b)–3(e)). This result indicates that the epitopes of the MAbs raised against yeast-derived SBV N protein are not sensitive to denaturation. Recently, it was reported that the MAb 2C8 generated against recombinant SBV N protein expressed in* E. coli* is reactive with virus-derived N protein in the lysates of SBV-infected Vero and BHK cells by western blot [32].

### 3.4. MAb Reactivities with SBV-Infected Cells

**Figure 4:**
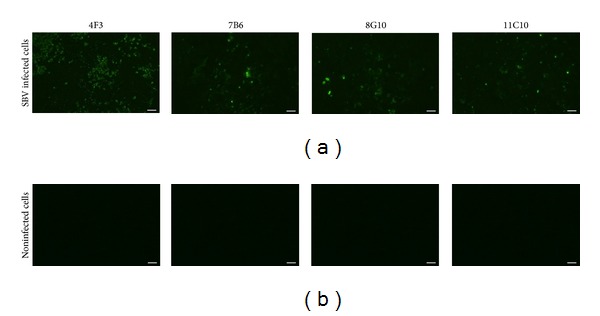
Fluorescence microphotographs showing the reactivity of the MAbs with BHK cells infected with SBV BH80/11 strain (a). Noninfected BHK cells were used as a negative control (b). Hybridoma culture supernatants were used at a dilution of 1 : 10. MAbs codes are indicated on the top of each picture. Scale bar: 100 *μ*m.

To prove whether the MAbs raised against recombinant SBV N protein recognize viral N protein, the reactivities of the MAbs were tested by an immunofluorescence analysis using BHK cells infected with SBV BH80/11 initially used for the isolation of SBV N protein gene. All MAbs were reactive with SBV-infected BHK cells, although the intensity of the immunostaining was differently dependent on MAb clone (Figure 4(a)). No immunoreactivity of the MAbs with noninfected BHK cells used as a negative control was observed (Figure 4(b)).

The immunofluorescence assay data confirm that the MAbs raised against yeast-expressed SBV N protein recognize viral nucleocapsids, which is an additional evidence on the antigenic similarity between yeast-expressed N protein and virus-derived N protein. The MAbs against SBV are of special interest, as they could be used for the development of simple and rapid laboratory diagnostic assays for direct virus detection in biological specimens.

## 4. Conclusions

Yeast expression system was successfully used to produce recombinant SBV N protein. Purified recombinant SBV N protein was reactive with SBV IgG-positive cow serum specimens collected in Lithuania. For the first time, the circulation of SBV virus in Lithuania was demonstrated. Immunization of mice with SBV N protein resulted in four MAbs that were reactive with SBV-infected cells. The reactivity of recombinant N protein with SBV-specific IgG in cow sera as well as the ability of the MAbs raised against recombinant SBV N protein to recognize native viral nucleocapsids confirms that yeast-expressed SBV N protein resembles native virus in regard of antigenicity and morphology. In summary, yeast-expressed SBV N protein and newly developed SBV-reactive MAbs may provide useful reagents for diagnostics and seroprevalence studies of SBV infection.

## Figures and Tables

**Table 1:** Characterization of the MAbs raised against yeast-derived SBV N protein.

Clone	Subtype	*K*_*d*_, M
4F3	IgG2b	1,39 × 10^−10^ ± 2,9 × 10^−11^
7B6	IgG1	3,77 × 10^−10^ ± 8,9 × 10^−11^
8G10	IgG1	3,58 × 10^−10^ ± 9,3 × 10^−11^
11C10	IgG1	2,47 × 10^−9^ ± 2,4 × 10^−10^
